# Swallowed dental bridge causing ileal perforation: a case report

**DOI:** 10.1186/1757-1626-1-392

**Published:** 2008-12-12

**Authors:** Farhan Rashid, John Simpson, G Ananthakrishnan, Gillian M Tierney

**Affiliations:** 1 Department of Surgery, Derby City General Hospital, Uttoxeter Road, Derby, DE22 3NE, UK; 2 Department of Surgery, Nottingham University Hospitals, Queens Medical Centre, Nottingham, NG7 2UH, UK

## Abstract

We report the case of a 53 year old gentleman who had accidentally swallowed his dental bridge. One week following this he experienced a sudden onset of generalised abdominal pain and underwent laparotomy. At operation he was found to have a distal ileal perforation and an ileocaecal resection was performed. Although most swallowed foreign bodies pass through the gastrointestinal tract without problem, serious complications including intestinal perforation can occur.

## Background

Swallowed foreign bodies are not an uncommon occurrence but fortunately most pass through the gastrointestinal tract without complication. We present a rare case of a swallowed dental bridge which led to intestinal perforation necessitating an ileocaecal resection.

## Case presentation

**Figure 1:**
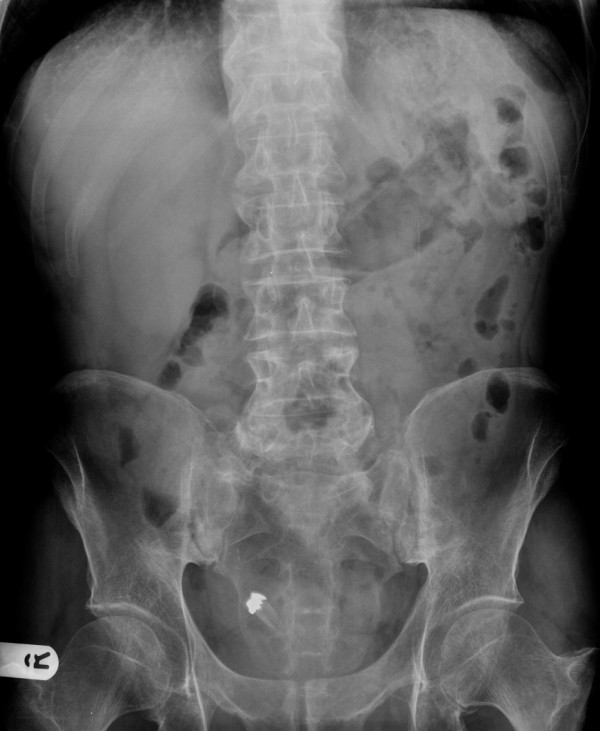
Abdominal x-ray showing the foreign body in right iliac fossa.

A 53 year old gentleman presented via Accident and Emergency department with a 3 day history of colicky, intermittent lower abdominal pain. There were no other gastrointestinal symptoms apart from that he had swallowed his dental bridge seven days previously. General and abdominal examination was unremarkable. Investigations revealed a raised white cell count at 16.5 × 10^9 ^cells/l and plain supine abdominal x-ray confirmed the presence of a foreign body over the right lower quadrant (figure 1).

**Figure 2:**
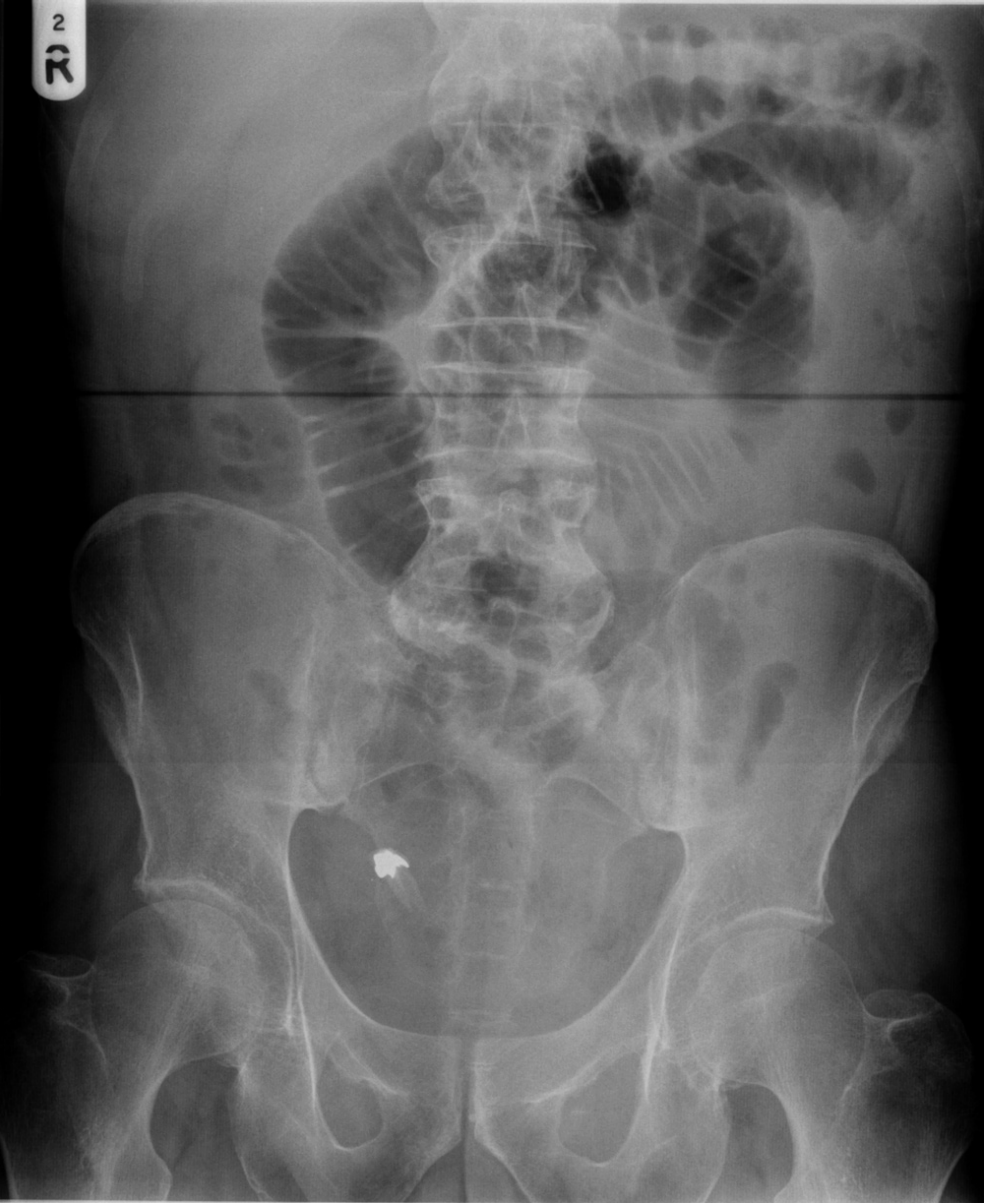
Abdominal x-ray showing denture causing small bowel obstruction.

He was admitted for observation and 24 hours following admission he complained of a sudden onset of increased, generalised abdominal pain. At this time he became pyrexic (38.5°C) and tachycardic (pulse rate = 110 beats per minute) and examination revealed a distended abdomen with marked tenderness in the right iliac fossa. A further abdominal x-ray demonstrated features of small bowel obstruction (figure 2).

**Figure 3:**
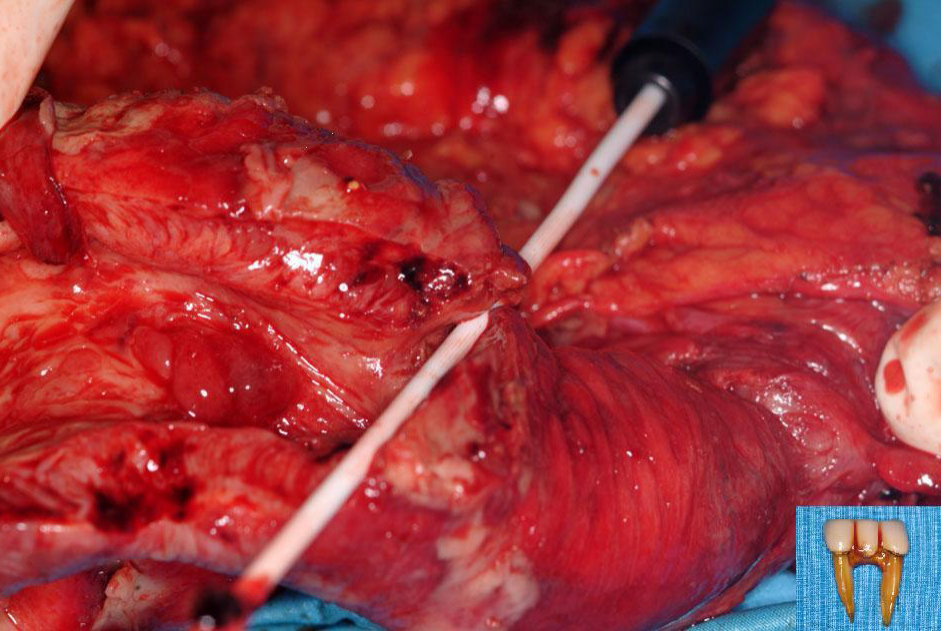
Resection specimen showing ileal perforation. Inset – retrieved dental bridge.

A laparotomy was performed which showed free pus and fluid within the peritoneal cavity. There was a distal ileal perforation the point of dental bridge impaction surrounded by a phlegmonous mass involving omentum, ileum and caecum. An ileocaecal resection (figure 3) and thorough peritoneal lavage were performed and the postoperative course was uneventful.

## Discussion

The accidental or deliberate ingestion of foreign bodies is not an uncommon occurrence although fortunately most pass through the gastrointestinal tract without problem. The typical zones of foreign body impaction areas of relative anatomical or physiological narrowings such as the upper and lower oesophageal sphincters, pylorus, duodenum, ileocaecal valve, appendix, sigmoid colon and anus. Although wearing dentures can lead to loss of sensation within the oral cavity and increase the likelihood of accidental ingestion of foreign bodies[1], swallowing dental prosthesis themselves can lead to problems and previously reported cases include tracheoesophageal fistula[2] and perforation of the colon[3]. However, due to the wide variety of dental prostheses it is difficult to advocate a management to cover all scenarios but as this case suggests, serious complications requiring operative intervention can occur.

## Conclusion

Although most swallowed foreign pass through the gastrointestinal tract without problem serious complications including intestinal perforation can occur.

## Consent

Written informed consent was obtained from the patient for publication of this case report and accompanying images. A copy of the written consent is available for review by the Editor-in-Chief of this journal.

## Competing interests

The authors declare that they have no competing interests.

## Authors' contributions

Mr. F Rashid and Mr. J Simpson reviewed the literature and wrote the manuscript. Mr. G Ananthakrishnan attended the patient whilst on the ward. Miss G Tierney and Mr. J Simpson performed the operation. All authors contributed intellectual content and have read and approved the final manuscript.
